# A qPCR-Based Screening Platform for Exploratory Assessment of Phage Training Outcomes in *Enterobacter cloacae* and *Stenotrophomonas maltophilia*

**DOI:** 10.3390/v18060624

**Published:** 2026-05-29

**Authors:** Ghadeer Jdeed, Vera Morozova, Valeria Fedorets, Tatiana Ushakova, Lina Al Allaf, Igor Babkin, Nina Tikunova

**Affiliations:** Laboratory for Molecular Microbiology, Institute of Chemical Biology and Fundamental Medicine SB RAS, 630090 Novosibirsk, Russia; morozova@1bio.ru (V.M.); f.valeriya41@gmail.com (V.F.); ushakova@1bio.ru (T.U.); allaflina@1bio.ru (L.A.A.); i_babkin@mail.ru (I.B.)

**Keywords:** real-time PCR, phage training, phage therapy, phage cocktails, bacteriophage, experimental evolution, qPCR, high-throughput screening, host-range expansion, co-evolution

## Abstract

Bacteriophages (phages) represent promising therapeutic agents. Their clinical use is challenged by the rapid rise of resistant bacterial clones. To overcome this problem, phages can be trained in vitro to improve their ability to cope with the possible resistance that may arise. Here, we co-evolved phages with their hosts under different conditions and assessed their ability to infect an adapted bacterial panel using qPCR. The co-evolution experiment yielded a panel of bacterial clones adapted either to a phage, a competing phage, or a cocktail of both. Phages were adapted either in the continuous presence of an evolutionarily naïve host, in a cocktail with a competing phage, under both conditions, or under neither condition. We assessed each resulting phage for its ability to infect evolved bacterial clones in the panel we created, using qPCR for preliminary high-throughput assessment. This allowed us to evaluate 500 phages–bacteria interactions. Overall, qPCR-detected phage production following infection of different bacterial clones improved to varying degrees when evolutionary naïve hosts were added during the training. However, the screening suggests that optimal training conditions are phage-specific. For *Enterobacter cloacae* phages EC151 and EC152, the broadest increase in qPCR-estimated phage production in our experiments was observed when a competing phage and/or an evolutionarily naïve host was included during adaptation. For *Stenotrophomonas maltophilia* phages StM171 and StenM174, the presence of an evolutionarily naïve hosts appeared beneficial in both replicates; co-adaptation with a competing phage led to a complete loss of StM171 infectivity in both experiments but benefited StenM174. Phages passaged for ten passages consistently infected a broader range of bacterial clones than those sampled after five passages. Sequencing of eight EC152-derived phages identified recurring mutations in a transcriptional regulator, and in some cases, in baseplate and tail fiber genes.

## 1. Introduction

The rise of antibiotic resistance has made bacteriophages (phages) an increasingly promising alternative for therapeutic applications [1]. Phages are specific, widely available, and can be utilized as alternatives to, or in addition to, antibiotics [2]. However, phage therapy faces several challenges, one of which is the rapid emergence of bacterial resistance during treatment [3]. To address this issue, phage therapy is often administered as cocktail of different phages targeting the same bacterial strain [4]. This approach aims to minimize the likelihood of bacteria developing resistance to the various phages used, as developing resistance to each of them can impose greater fitness costs on the bacteria [5].

While the use of cocktails can help mitigate the emergence of bacterial resistance, identifying multiple phages specific to the target pathogenic bacterial strain is not always feasible [6]. An alternative approach to combat bacterial resistance is the pre-training of phages. This process involves co-evolving phages with the target bacteria by incubating them together over several passages [7]. The goal of this training process is to produce phage populations already adapted to the phage-resistant bacteria that are likely to emerge during phage treatment.

This straightforward process can be modified to improve the characteristics of the resulting phages. In recent years, various factors have been explored [8,9]. For instance, research has demonstrated that adding evolutionarily naïve bacteria allows phages to explore novel mutations, which can help them evade bacterial resistance [10,11]. This may lead to an expansion of the phage host range, albeit at the expense of growth rate [12]. Other studies have indicated that the presence of competing phages in co-evolution experiments may have either antagonistic or synergistic effects on the resulting phages [8,13]. Competing phages may inhibit bacterial growth more effectively and help phages evade CRISPR-Cas immunity [14]; however, they can also accelerate the emergence of resistant bacteria or reduce phage efficiency, particularly if they compete for the same attachment sites [15]. In addition, other factors have been studied over the past twenty years [16]. Selecting the appropriate factors presents a challenge, as identifying optimal conditions and monitoring the lytic activity of the resulting phages against emerging bacterial populations can be labor-intensive and time-consuming.

Traditional plaque assays, while standard, are low-throughput and manually demanding when screening numerous phages–bacteria combinations. As a potential solution, real-time PCR (qPCR) serves as a valuable tool for addressing several challenges associated with phage studies. It enables researchers to scale investigations of various phage characteristics [17]. However, it does have limitations, primarily related to its inability to distinguish between living organisms and DNA remnants [18,19].

Here, we present a qPCR-based screening approach, supported by a set of controls, to evaluate phages–bacteria interactions and identify the most effective phage training strategy and the optimal phage population obtained after ten passages.

We tested four training strategies across four different phages in two experiments (biological replicates) over five and ten passages: (A) training the phage with its bacterial host; (B) introducing naïve hosts to maintain a source for phage propagation and prevent extinction, (C) incorporating a competing phage to promote broad adaptation; and (D) combining naïve hosts and a competing phage. 

We studied phages that infect two different bacterial species, *Stenotrophomonas maltophilia* and *Enterobacter cloacae*. We aimed to model a realistic phage-training scenario in which phages are urgently needed for therapy, and phages already available in a phage bank must be adapted for downstream use rather than isolating new ones from nature. To do this, we used phages with varying lytic abilities and included a temperate phage to examine whether its presence could influence the evolution of the other phages. We asked whether any of the four phages would show directional trends toward maintained or broadened qPCR-estimated phage production under these training scenarios. While prior work has explored individual training factors [10], we focused on identifying consistent patterns across phages and on using a qPCR-based screening platform as a scalable tool for such phage-training assessments. In addition to phenotypic screening, genomic analysis of evolved populations of phage EC152 was conducted.

## 2. Materials and Methods

### 2.1. Bacterial Strains and Phages Used and Growth Conditions

Bacterial strains used in this study were obtained from the Collection of Extremophilic Microorganisms and Type Cultures (CEMTC) of the Institute of Chemical Biology and Fundamental Medicine, Siberian Branch of the Russian Academy of Sciences (ICBFM SB RAS). The following bacteria were used: *E. cloacae* strain CEMTC 2064 and *S. maltophilia* strain CEMTC 2355. The bacteria were cultivated in Luria–Bertani broth (Thermo Fisher Scientific, Waltham, MA, USA) aerobically at 37 °C. Bacterial growth was monitored by measuring optical density at 600 nm (OD600), an OD600 of 1.0 corresponded to 8 × 10^8^ cells/mL. The following phages from the CEMTC ICBFM SB RAS were used: *Enterobacter* phage EC151, a weakly lytic phage [20], *Enterobacter* phage EC152, a lysogenic phage, both infecting *E. cloacae* CEMTC 2064. In addition, *Stenotrophomonas* phage StM171, a weakly lytic phage [21], and lytic *Stenotrophomonas* phage StenM174 [22], both specific to *S. maltophilia* CEMTC 2355 were used. The characteristics of these phages are summarized in Table 1. And the genomic, phylogenetic and biological characteristics of EC152 are described in more detail in Supplementary File S1.

### 2.2. Adapting qPCR for Characterization of Phage Biological Properties and Phage Infection Yields

Phage infection efficiency was assessed by measuring changes in the number of phage particles after incubation with the bacterial host. To assess infection efficiency, qPCR was performed according to the protocol presented in Table 2, using oligonucleotides targeting unique genes of the phages under study (Table 3). Raw fluorescence data were processed using the regression algorithm in CFX Manager software 3.1.

Phage samples used for qPCR were prepared as follows: 50 µL of each sample was incubated at 100 °C for 30 min to disrupt the capsids. An equal volume of chloroform was then added, the mixture was vigorously vortexed for 10 s, and it was centrifuged at 14,000 rpm for 5 min. Five microliters of the supernatant containing phage DNA was collected for qPCR analysis. If necessary, the extracted DNA was diluted 5-fold with sterile water to reduce the potential inhibitory effect of bacterial components.

**Figure 1:**
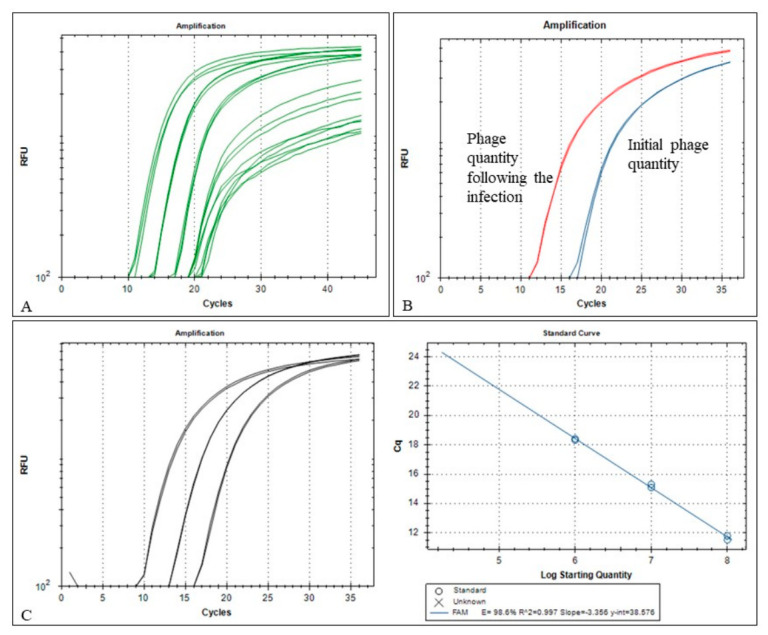
Controls used in the study and an example of detecting limit of detection: (**A**) Serial dilutions of a phage sample to determine the detection limit. (**B**) Control #1 is used to estimate the increase in the quantity of phage particles after infection of bacteria. (**C**) Control #2, is used to approximately determine the titer of the amount of phage by comparison with a serial dilution of a phage sample with a known number of PFU/mL.

Serial 10-fold dilutions of the phage were used to construct a standard curve. The detection limit was set at the lowest concentration with a Cq value above background noise and within the linear range (~100% efficiency; ΔCq ≈ 3.3 per log dilution; Figure 1A). Two types of controls were used in each PCR run. Controls #1 were used to assess the ability of the phage to infect bacteria: the phage Cq value after 24 h of incubation with bacteria was compared with its Cq value before infection. This was done by including a phage sample diluted to the same initial concentration as the phage used to infect the bacteria (Figure 1B). Controls #2 were used to quantify phage particles in the samples and determine the efficiency of the PCR reaction; three serial dilutions with a known number of PFU/mL of the tested phage were included as standards in each run (Figure 1C).

Reproducibility of the results was evaluated by calculating the mean and standard deviation of the Cq values obtained for control samples #2 for each of the four tested phages across different runs. We also examined the range of reaction efficiency and the slope across runs for the same sample.

### 2.3. Co-Evolution Experiment and Studied Scenarios

Using the optimized qPCR method, we investigated how phage adaptation affected the ability of phages to infect clones derived from their original host. We studied two phages infecting *E. cloacae* CEMTC 2064, EC151 and EC152, and two phages infecting *S. maltophilia* CEMTC 2355, StM171 and StenM174.

Phage adaptation was achieved by passaging them with their respective hosts. This was done in two independent biological repeats lasting 10 passages under four different scenarios. The scenarios were as follows (Table 4):

1. Scenario A: phage co-evolution with the bacterial host; the experiment was performed for all four phages, each of which was passaged separately with the host strain.
2. Scenario B: similar to scenario A, but with daily addition of an evolutionarily naïve (ancestral) host (OD600 = 0.3); for phages EC151 and EC152, *E. cloacae* CEMTC 2064 was added, and for phages StM171 and StenM174, the ancestral *S. maltophilia* strain CEMTC 2355 was added.
3. Scenario C: co-evolution of phage with host in the presence of a competing phage; StenM174 and StM171 were prepared as a cocktail at equal titers before the start of the co-evolution experiment and passaged together, and the same was done for EC151 and EC152.
4. Scenario D: A combination of scenarios B and C; the same cocktails were prepared with daily additions of evolutionarily naïve bacteria.

**Figure 2:**
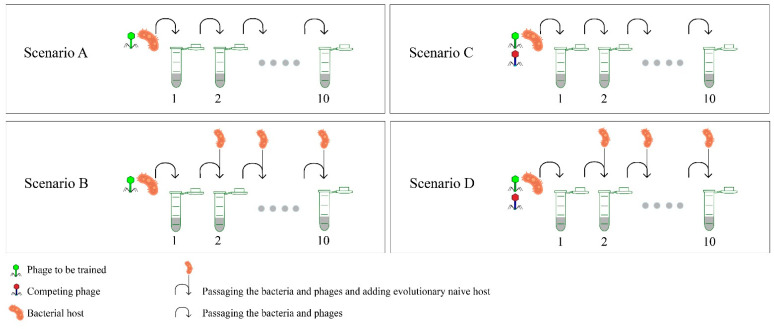
General scheme of the four scenarios under which the phages were adapted, the numbers indicate the passage.

Each experiment began with growing bacteria to an OD600 of 0.3, followed by the addition of phages at an MOI of 0.1. The total volume of the phages–bacteria mixture was 700 µL; the suspension was incubated at 37 °C with shaking at 180 rpm for 24 h. At each passage, 35 µL of this mixture was transferred to a new tube containing 665 µL of LB medium. For scenarios B and D, an additional 10 µL of naïve bacteria grown the same day to an OD600 of 0.3 were added to each passage, and the tubes from the previous passage were then stored at 4 °C (Figure 2).

Phages and bacteria collected after the 5th and 10th passages were separated by centrifugation at 5000 rpm for 5 min. Bacterial pellets obtained after the 5th and 10th passages in scenarios A and C were resuspended and grown on LB agar in Petri dishes. The mixture of individual colonies was transferred to new Petri dishes three additional times and analyzed by PCR to confirm the absence of residual phages. Bacteria from scenarios B and D were not used for further analysis because evolutionarily naïve bacteria added at each passage.

The centrifuged suspension containing the phage particles was transferred to new centrifuge tubes. A portion of the suspension was used to isolate phage DNA, and the number of phage genomes was determined using qPCR by comparison with control samples #2. Thus, for each phage, eight different phage populations were obtained by the end of each experiment (adapted according to scenarios A, B, C, and D after passages 5 and 10), along with six bacterial populations (adapted against one of the phages, adapted against a competing phage, and adapted against a mixture of both phages, after passages 5 and 10). These phage and bacterial populations were used in the subsequent experiment in addition to the ancestral phages and bacteria.

### 2.4. Assessing Phage Production by qPCR

Passaging a bacterial population with two different phages, either individually or in a cocktail, resulted in different bacterial clones. To assess the success of phage adaptation, we examined two metrics: (1) whether the adapted phage population could infect all variable bacterial clones in addition to the original host; and (2) whether an adaptation scenario increased phage infection efficiency compared to the ancestral phage.

To answer these questions, a portion of the suspension volume containing the phage populations obtained in each scenario (A, B, C, D) after the 5th and 10th passages, as well as the original phage, was used to infect the bacterial populations obtained during the experiment from scenarios A and C after the 5th and 10th passages, in addition to the original bacterial host. Bacterial cultures were grown to an optical density (OD600) of 0.3, and phages were added at a constant multiplicity of infection (MOI = 0.1). Incubation was performed overnight without shaking in 96-well plates at 37 °C in LB medium. Phage DNA samples for real-time PCR were then prepared as described previously (Section 2.2). Thus, more than 500 phages–bacteria interactions were studied using qPCR, with each qPCR run including two technical replicates.

qPCR-estimated phage production was calculated as the ratio of the number of phage genomic particles after bacterial infection (calculated as titer) to the known titer of the control sample #1, which represents the same phage before the adaptation experiment, this ratio was expressed as log10 phage particles/mL. A significant increase in the quantity of phage particles after infection was considered as an indicator of phage ability to infect bacteria. The threshold value established for determining the ability of an adapted phage to infect bacteria was 0.5, which represents a 3-fold increase in the number of phage particles after infection of bacteria compared to the initial concentration, this threshold was based on the average qPCR-detected phage production of the ancestral phages when infecting the ancestral bacterial hosts.

$$qPCR\;detected\;phage\;production=\log_{10}\frac{quantity\;of\;phage\;particles\;after\;infection}{quantity\;of\;phage\;particles\;before\;infection\;(control\;\#1)}$$

The qPCR-based estimate of the increase in phage production was calculated by comparing the ability of the adapted phage to infect a bacterial population with the ability of the ancestral phage to infect the same bacterial population. For this study, we considered only increases greater than 1 (i.e., a 10-fold increase in the number of phage particles infectable by the adapted phage compared to the initial phage, or 3.3 Cq according to qPCR). We also considered an adapted phage to have enhanced particles production against a bacterial population if the ancestral phage was unable to infect that population, while the adapted phage was able to do so.

$$qPCR\;detected\;increase\;in\;phage\;production=\frac{Adapted\;phage\;detected\;production}{Ancestral\;phage\;detected\;production}$$

Following the calculations, the observed outcomes are reported for each replicate individually.

### 2.5. DNA Isolation and Sequencing of Adapted Phages

Adapted populations of phage EC152 were propagated until a high titer of 10^10^ PFU/mL was obtained. Phage DNA was then isolated as described previously [20] and fragmented using Covaris S2 Focused-ultrasonicator (PerkinElmer U.S. LLC, Washington, DC, USA) with a target fragments length of approximately 500 bp. The fragmented DNA was purified using VAHTS DNA purification beads (Nanjing Vazyme Biotech Co., Ltd., Jiangsu Province, China) and used for preparation of pair-end libraries with the NadPrep DNA Library Preparation Kit v2 and NadPrep Universal Stubby Adapter (UDI) Module (Nanjing Biotechnology Co., Ltd., Nanjing, Jiangsu Province, China). DNA sequencing was performed using the GeneMind FASTASeq 300 sequencer and FCH flow cell PE150 (GeneMind Biosciences Co., Ltd., Shenzhen, China).

The reads obtained were filtered by quality using Trimmomatic v.0.39 [23] (accessed on 20 February 2026). The genomes were assembled de novo using SPAdes Genome Assembler v.3.15.5 (accessed on 20 February 2026) [24].

### 2.6. Bioinformatic Analysis of Phages’ Genomes

The assembled EC152 genomes of the adapted populations were aligned manually to the reference genome of the ancestral phage EC152 (GenBank PP681140) using UNIPRO UGENE software 53.1 [25]. Additionally, the resulting sequences of EC152 were compared to the ancestral phage using BLASTn (2.17.0) [26], the proteins containing mutations were further analyzed using HHpred [27] and PSIPRED [28].

## 3. Results

### 3.1. Optimization of Sample Preparation with Phage DNA as a Template for qPCR

The reproducibility of the qPCR data was confirmed by analyzing the same phage samples in control #2 across several runs. Apart from phage StM171, reaction efficiencies ranged from 95% to 105%, and the maximum standard deviation and Cq value variation for the same samples across runs were considered acceptable (≤0.3) (Table 5). No amplification of the negative controls (water) was observed.

Approximate phage titers were estimated by comparing the Cq values of diluted samples with known titers from the same runs determined by the double agar method. The detection limit of phages was determined, and we found that real-time qPCR could reliably quantify the concentrations of phages down to ~10^4^ PFU/mL, with sensitivity decreasing below 10^3^ PFU/mL.

### 3.2. Results of Adaptation of Stenotrophomonas Phages StM171 and StenM174

To assess the success of phage adaptation under different co-evolution scenarios, we evaluated two key metrics: (1) the ability of evolved phage populations to infect a panel of bacterial clones adapted to the phage itself, to a competing phage or to a cocktail of both phages; and (2) whether any adaptation scenario increased infection efficiency compared to the ancestral phage. Although the results were phage specific and showed variation between the two experiments, possibly due to stochastic mutation introduction, several general trends were observed regarding the conditions that favored the emergence of generalist phages, capable of infecting most, if not all, bacterial clones. These trends were inferred from the increase in phage particles after infection compared with the quantity of phage particles before infection. Phages obtained after ten passages were consistently more effective than those obtained after five passages. For *S. maltophilia* phages, the presence of an evolutionarily naïve host was more beneficial than co-adaptation with a competing phage.

**Figure 3:**
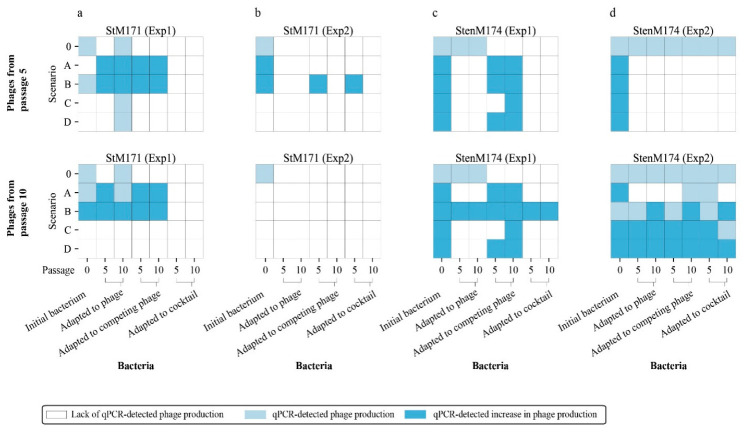
Results of passaging of phages StM171 experiment 1 (**a**) and experiment 2 (**b**), and StenM174 experiment 1 (**c**) and experiment 2 (**d**).

Among the four phages studied, StM171 showed a marked loss of qPCR-detected infectivity across most evolved bacterial clones. In both experiments, adapting StM171 with the competing phage StenM174 (scenarios C and D) completely prevented detectable phage production against all bacterial clones tested (Figure 3a,b). The one exception occurred when StM171 was adapted alone (scenario A) or in the presence of naïve host (scenario B) for ten passages. In one of these two experiments, these conditions yielded phage populations capable of infecting most bacterial clones, including the ancestral bacterial strain and the clones adapted to StM171 or StenM174 separately for five or ten passages. However, these successful populations could not infect any bacteria that adapted to a cocktail of both phages (Figure 3a).

StenM174 showed greater adaptability than StM171 under the conditions tested. Although resistance to StenM174 still arose in the bacterial clones across both experiments, longer adaptation (ten passages) in the presence of a naïve host (scenario B) yielded phage populations that infected all the tested bacterial clones in both experimental replicates. StM171 had beneficial effects on the adaptation of StenM174 in two ways. First, in the first experiment, an cross-sensitization was observed, where bacteria evolved against StM171 became sensitive to StenM174 (Figure 3c). Second, in the second experiment, adapting StenM174 in a cocktail with StM171, with or without a naïve host (scenarios C and D), produced phage populations capable of infecting all bacterial clones (Figure 3d). Regarding enhanced phage production, scenario B (adapting the phage alone in the presence of an evolutionarily naïve host) in experiment one, and scenario D (adapting the phage with the competing phage StM171 in the presence of an evolutionarily naïve host) in experiment two were the only ones in which StenM174 populations displayed an increase in genome copies relative to the ancestral phage against all bacterial clones. The detailed values of the increase in phage quantity following the infection are provided in Table S1.

### 3.3. Results of Adaptation of Enterobacter Phages EC151 and EC152

**Figure 4:**
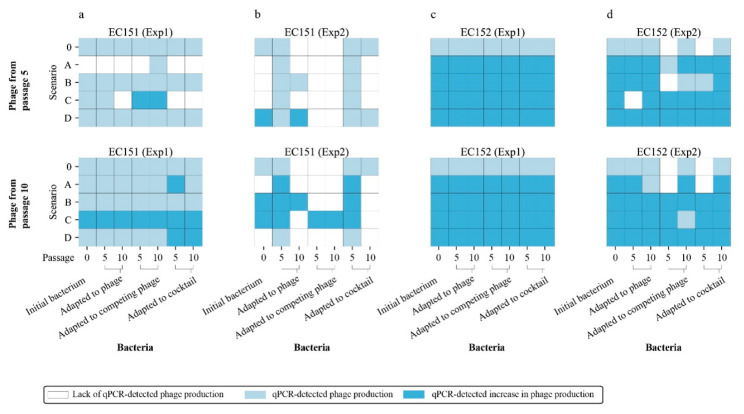
Results of passaging of phages EC151 experiment 1 (**a**) and experiment 2 (**b**), and EC152 experiment 1 (**c**) and experiment 2 (**d**).

In contrast, for the *E. cloacae* phages, both the naïve host and the competing phage promoted the emergence of generalist phages with increased phage production relative to the ancestral phages. For phage EC151 after ten passages, all adapted populations infected every bacterial clone in the first experiment; in the second experiment, scenario C produced phages that infected five of seven clones and showed increased genome yields (Figure 4a). In the second experimental repeat, scenario C again produced the most effective phages among the tested scenarios; the resulting phages showed increase in phage quantities after infecting five of seven bacterial clones and produced more phage particles than the ancestral phage (Figure 4b). Scenario B (adaptation with a naïve host) was the next most successful, yielding a phage population able to infect all bacterial clones in the first experimental repeat and four of seven clones in the second experiment, although enhanced phage production was observed only in the second experiment (Figure 4).

Phage EC152 displayed the broadest qPCR-estimated production profile of the four phages. After ten passages, EC152 populations adapted in scenario A (no naïve host and no competing phage) were able to infect most of the bacterial clones in both experiments. Furthermore, populations from scenarios B, C and D (presence of a naïve host, a competing phage, or both) were able to infect all obtained bacterial clones in both repeats, with enhanced phage production compared to the ancestral phage (Figure 4c,d).

### 3.4. Results of Sequencing of the EC152 Adapted Populations

Since EC152 was the only phage with consistently enhanced outcomes across all four scenarios after ten passages, we examined mutations in the genome of the adapted EC152 populations. Four populations obtained after 10 passages were sequenced from the two experimental repeats. Complete genome sequencing with >200× coverage of EC152 populations from the two independent experiments after 10 passages revealed that genomes were largely (>99%) identical to the reference genome of the ancestral phage (GenBank PP681140), with seven SNPs identified by BLASTn (version 2.17.0) alignment. The mutations included three nonsynonymous mutations within ORFs and four intergenic mutations. The distribution of the mutations among the phages varied: 6/8 of the phages included the intergenic mutations, and 7/8 included the mutation P14T in the transcriptional regulator protein. Only phages trained in the presence of the competing phage EC151 (scenario C and D) in the second experiment included additional mutations: T26A in the baseplate protein and G342R in the tail fiber protein (Table 6).

**Figure 5:**
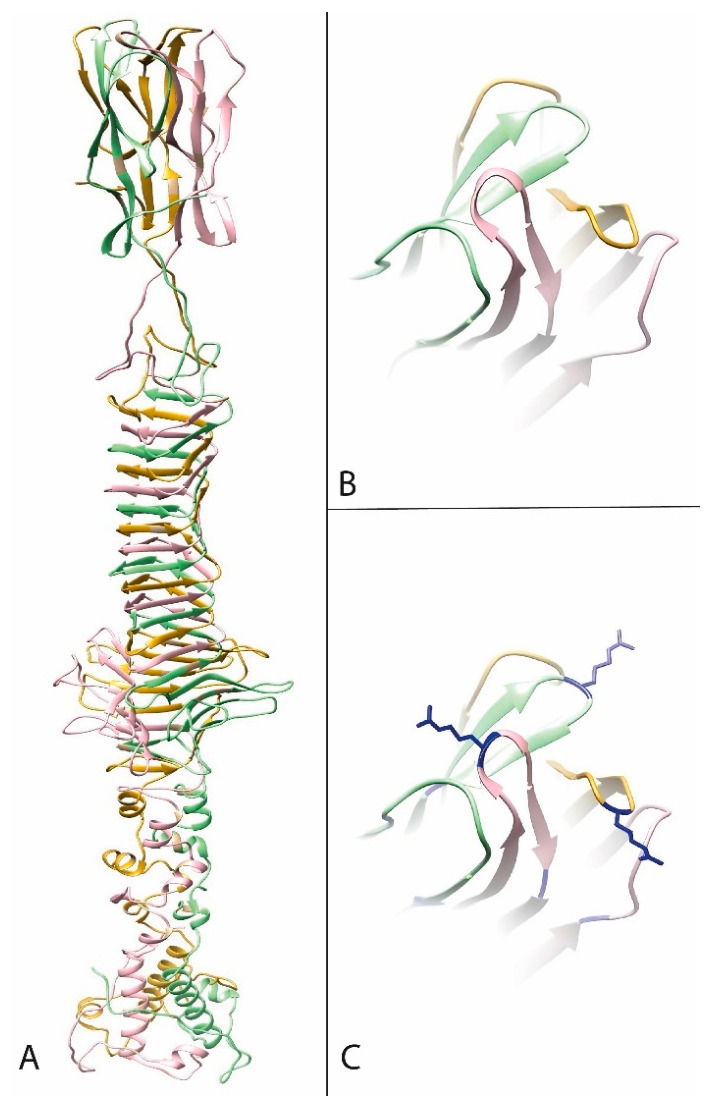
Ribbon representation of the predicted 3D structure of the tail fiber protein trimer of EC152; monomers are marked in green, rose and yellow. (**A**) Initial protein; N- and C-ends are noted. (**B**) C-terminal fragment of the initial protein. (**C**) C-terminal fragment of the mutated protein; arginine residues are marked in blue. The molecular coordinates of the predicted 3D structures were rendered using the UCSF chimera molecular visualizer, version 1.15.

The P14T mutation could affect DNA-binding affinity or promoter recognition, thereby potentially affecting transcription patterns, lytic gene expression or other functions under evolutionary pressure. The T26A substitution removes a hydroxyl group and slightly changes local polarity and packing of the protein; this could affect folding stability, interactions with neighboring baseplate subunits, or how the tail fiber docks. However, we speculate that it is less likely to change receptor binding, compared to the G342R substitution, which replaces the small, neutral glycine residue with a bulky, positively charged arginine residue (pKa ~12.5) (Figure 5).

Finally, the intergenic mutations were co-occurring, two intergenic mutations, PP681140: 4 C->T and PP681140:23 G->T, were located between the CDS of hypothetical proteins WZX10556.1 and WZX10819.1, and the other two intergenic mutations, PP681140:11597 C->T and PP681140:11603 C->G were located between the CDS with hypothetical functions WZX10585.1 and WZX10585.1.

## 4. Discussion

Phage–bacteria co-evolution is a process that can be used to enhance the therapeutic potential of phages, especially when addressing resistance after administration. We tested phage adaptation across four scenarios: (A) phage alone, (B) phage plus a naïve host, (C) phage and a competing phage without naïve host, and (D) phage and a competing phage plus a naïve host. In previous studies, the addition of a naïve host and a competing phage was found to be beneficial in some cases [29,30,31]. Adaptation was carried out over 10 passages, as 10 passages is a practical duration for application purposes and for training phages for therapy. Adapted phages and bacterial population were tested after the fifth and tenth passages against the bacterial panels evolved under single phage or two phages’ pressures.

In our two biological replicates, adding an evolutionary naïve host and extending adaptation to 10 passages was associated with broader adaptability for all four phages. For EC151 and EC152, the presence of a competing phage or a naïve host trended toward increase in phage particles measured in most tested conditions. These phages perhaps benefitted from their encoded counter anti-phage defense systems to maintain infectivity, namely preQ0 modification pathway in EC151 [20] and NAD+ reutilization pathway in EC152.

In contrast, StM171 proved refractory to adaptation under most conditions tested here, especially in the presence of the competing phage StenM174. The poor performance of StM171 could be associated with the absence of its own DNA and RNA polymerases, or the high evolutionary pressure imposed by StenM174, given its higher lytic ability and faster adsorption time. The only instance in which StM171 adapted was in the presence of an evolutionary naïve host in scenario B; however, this result was not replicated. StenM174 benefitted from the presence of an evolutionarily naïve host, and cross-sensibilization was observed in one experiment, where bacteria resistant to StM171 became more vulnerable to StenM174. This result is similar to the collateral sensitivity in *Klebsiella* when bacteria adapted to one phage by losing the capsular polysaccharide, which increased their vulnerability to LPS-binding phages [13]. It is worth noting that the experiments with the best adaptation outcomes involved ancestral phages that were discovered to be able to infect most, if not all, of the bacterial panel. This could reflect the initial broad infectivity of the ancestral phages against clones derived from the bacterial host, or it may indicate that fitness costs prevented the bacterial clones from developing complete resistance to the phage and all derived populations simultaneously.

The observed inter-replicate variability underscores the stochastic nature of experimental evolution. Differences in the initial pool of spontaneous mutations may dictate divergent adaptive trajectories, a limitation that can only be addressed with a substantially larger number of replicate populations. Despite this, the mutations observed in EC152 were consistent regardless of the scenario to which the phage adapted. In six of eight phages the same co-occurring intergenic mutations repeated, and in seven out of eight phages, the same mutation in the transcription regulator was observed. These mutations could be responsible for the high adaptation of EC152 under different stressors. A previous study showed that mutations in transcription regulators such as *cro* and *cI* genes in phage lambda influence the decision between lytic and lysogenic life cycles [11]. Additionally, intergenic mutations could also affect transcription, as in lambda-like phages, which have been shown to affect infection efficiency [12]. Other intergenic mutations were reported in *Klebsiella pneumoniae* phage ACE.3 [32] and *Listeria monocytogenes* phages following adaptation experiments [33]. Finally, the most frequently reported mutations in phages co-evolved to have an expanded host range were nonsynonymous mutations in the tail fiber and base plate gene. This pattern was reported in *K. pneumoniae* phages [32], *Bacillus subtilis* phage SPO1 [34], or *L. monocytogenes* phages [33]. In the present study, we observed mutations in the same genes in phage EC152-derived populations from scenario C and D in experiment 2. The mutation G342R in the C-terminal of EC152 tail fiber could potentially enhance electrostatic interactions with negatively charged host receptors, such as LPS, and possibly improve adsorption or host range, however this and the role of the other mutations we detected require functional validation that was outside the scope of the study.

This study is limited by the inclusion of only two biological replicates per condition, and therefore the results are presented as descriptive rather than statistically conclusive. However, these results showed recurring patterns related to the experiment duration and the addition of an evolutionary naïve host, regardless of the phage studied. Consequently, the results could be regarded as hypothesis-generating and descriptive, and they provide plausible directions for future work. We emphasize that the primary contribution of this work lies in demonstrating the feasibility and throughput of the qPCR-based screening platform, which can be used in larger, statistically powered evolutionary experiments and as a preliminary tool for identifying promising phage populations for therapy before more detailed in vivo testing. Lastly, two practical implications follow from this work: (1) a qPCR screening platform applied to a complex experimental design can efficiently screen 500 interactions in a short period of time (1–2 days), enabling faster selection of adapted phages for therapy; and (2) constructing bacterial panels for testing phages by evolving bacterial clones under diverse pressures, including single phage and cocktails, creates better screening tools mimicking in vivo mutants.

## Figures and Tables

**Table 1:** Overview of the phages’ features.

Phage	GenBank Accession Number	Genome Size (b.p)	Bacterial Host	Lytic Ability	Notable Accessory Genes	Host Range
EC151	MW464860	60753	*E. cloacae* CEMTC 2064	Decreases host cell titer by 0.5 order in 30 min	7-deazaguanine modification pathway against bacterial restriction modification systems	2 out of 8 *Enterobacter strains*
EC152	PP681140	148277	*E. cloacae* CEMTC 2064	Weakly lytic/temperate	NAD+ salvage system, RII locus defense system	2 out of 8 *Enterobacter* strains
StM171	MZ611865	44514	*S. maltophilia* CEMTC 2355	Decreases host cell titer by 0.5 order in 90 min	No DNA or RNA polymerase, DNA methyltransferase	5 out of 10 *Stenotrophomonas strains*
StenM174	OR729839.1	42956	*S. maltophilia* CEMTC 2355	Lytic, decreased host cell titer by 3 orders in 30 min	None	16 of 65 *Stenotrophomonas strains*

**Table 2:** qPCR protocol.

Temperature (C)	Time	Number of Cycles	Signal Reading
95	3 min	1	No
95	10 s	39 cycles	Yes
55	20 s		

**Table 3:** Oligonucleotides used for detection of the phages.

Phage	Oligo ID	Oligo Sequence 5’-3′	Target Gene Number (CDS)	Product Size (b.p.)
EC151	151_F	aggagcaggtagaaca	Hypothetical protein in the 7-deazaguanine modification pathway (QSL98919.1)	82
	151_R	gtcgtcgattgaaacttatcct		
	151_P	[FAM] ctggacaggcgccagcaatggat [BHQ1]		
EC152	152_F	cccagtgatcttatcgcaac	Hypothetical protein (WZX10802.1)	99
	152_R	atgatgcctatcgaactggt		
	152_P	[FAM] accactccagtgccagtacaacg [BHQ1]		
StM171	stm_stru_for	gcaggatccagtactacct	Structural protein (QYW06389.1)	118
	stm_stru_rev	aatgcaacgtcgatattcgt		
	stm_stru_probe	[FAM] ccgctgtgggtgccttccta [BHQ1]		
StenM_174	174_F	tcaggcttctacttcgttca	Murein transglucosylase-containing protein (WPK42350.1)	102
	174_R	cacttgtcattccacgtcag		
	174_P	[FAM] accgctgcgcagatcaagca [BHQ1]		

**Table 4:** Factors studied in each of the four scenarios.

Scenario	Daily Addition of Evolutionarily Naïve Bacteria	Presence of a Competing Phage from the Start of the Experiment
A	No	No
B	Yes	No
C	No	Yes
D	Yes	Yes

**Table 5:** Reproducibility of qPCR data and reaction efficiency for different phages across multiple runs.

Phage	Maximum Standard Deviation	Standard Deviation of Cq Between Runs	Slope	Reaction Efficiency Range (%)
StenM174	0.24	±0.10 cycle	−3.15	106.7–108
StM171	0.3	±0.62 cycle	−2.92	104.6–134
EC151	0.1	±0.12 cycle	−3.25	103–106
EC152	0.19	±0.15 cycle	−3.31	98.6–103

**Table 6:** Distribution of different mutations in EC152 populations obtained after 10 passages in two independent experiments.

Phage EC152	Co-occurring Intergenic Mutations 4 C->T; 23 G->T; 11597 C->T; 11,603 C->G	P14T Mutation in the Transcriptional Regulator WZX10670.1	T26A in the Baseplate Protein WZX10726.1	G342R in the Tail Fiber Protein WZX10735.1
Scenario A Exp #1	+	+	−	−
Scenario A Exp #2	+	+	−	−
Scenario B Exp #1	−	−	−	−
Scenario B Exp #2	+	+	−	−
Scenario C Exp #1	+	+	−	−
Scenario C Exp #2	−	+	+	+
Scenario D Exp #1	+	+	−	−
Scenario D Exp #2	+	+	+	+

## Data Availability

The original contributions presented in this study are included in the article/Supplementary Material. Further inquiries can be directed to the corresponding author.

## Supplementary Materials

The following supporting information can be downloaded at: https://www.mdpi.com/article/10.3390/v18060624/s1, File S1: phage EC152 characteristics; Table S1: increase in efficiency of infection.
