# Supplementary material for: A qPCR-Based Screening Platform for Exploratory Assessment of Phage Training Outcomes in Enterobacter cloacae and Stenotrophomonas maltophilia
Source: Viruses. 2026 May 29;18(6):624. doi: 10.3390/v18060624 (PMC13307863; doi:10.3390/v18060624)
Supplement: Supplementary file 1 [file viruses-18-00624-s001.zip › File S1. Phage EC152 characteristics.pdf]

Characterization of the E. cloacae EC152 phage

Phage EC152 was isolated from cattle manure. This phage forms turbid, poorly distinguishable plaques approximately 0.5 mm in diameter on a lawn of the host strain *E. cloacae* CEMTC 2064 (Fig. S1A). Transmission electron microscopy of phage EC152 virions revealed icosahedral heads 90-95 nm in diameter, connected to a long, contractile tail approximately 120 nm long. The morphology and size of the phage particles were consistent with the myovirus morphotype (Fig. S1B).

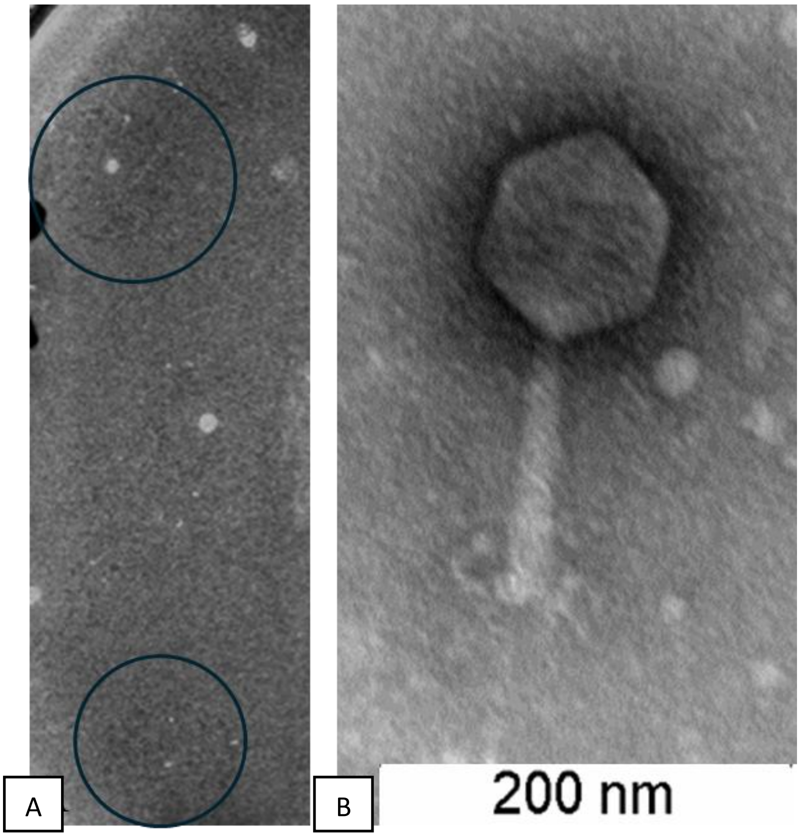

Figure S11Morphological properties of phage EC 152. A: Photograph of plaques formed by phage EC152 on a lawn of the host strain *E. cloacae* CEMTC 2064. B: Transmission electron micrograph of a particle of phage EC 152, negative staining with 1% uranyl acetate solution.

Because this phage forms very small, turbid plaques that are difficult to count, qPCR was used to assess phage infectivity, quantifying the amount of replicating phage DNA in the phage-bacteria culture. The rate of phage EC152 replication was similar to that of the host bacteria (Fig. S2A); the titer of bacteria infected with phage EC152 1.5 hours after infection was virtually identical to the titer of the control culture not infected with phage EC152, indicating that the phage replicates in parallel with bacterial division without obvious lysis (Fig. S2).

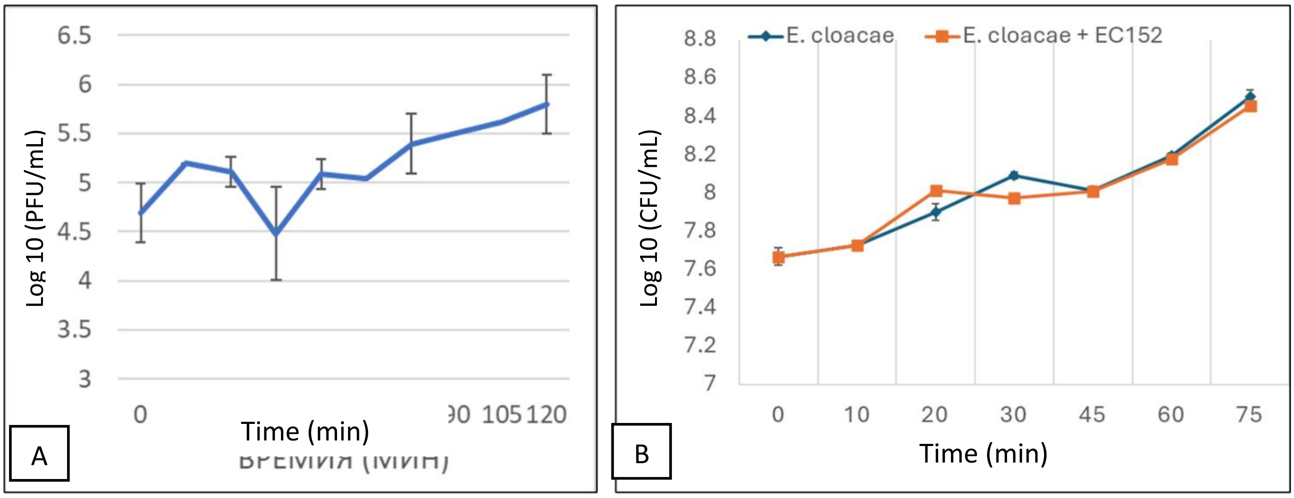

Figure S2. Biological characteristics of EC152 against *E. cloacae* CEMTC 2064. A) One-step growth curve of EC152 on the host strain *E. cloacae* CEMTC 2064. B) lysis kinetics, in which a bacterial culture incubated with phage EC152 (*E. cloacae* + EC152) was compared with an uninfected bacterial culture (*E. cloacae)*.

EC152 was found to be able to infect *only* 2 *E. cloacae* strains out of 8 *E. cloacae*, *E. hormaechei* and *E. xiangfangensis* strains tested.

Analysis of the EC152 genome revealed that it contains 148,277 bp, with 286 putative ORFs (Fig. S3). Functions were predicted for 51 of these ORFs, revealing a genome adapted to a temperate lifestyle. Key evidence for this includes the integrase and transposase-like protein genes, the latter suggesting a potential Mu-like mechanism for integration into the host genome [1]. EC152 phage may employ a defense strategy against the host bacterial immune system through the use of two distinct DNA methyltransferase genes, which likely protect its DNA by suppressing the activity of host restriction endonucleases. Metabolic adaptation is evident in the presence of a fully functional NAD+ reconstitution pathway (NAPR or NAD+ reconstitution pathway), consisting of the nicotinamide mononucleotide (NMN) transporter (PnuC) and nicotinamide nucleotide adenylyl transferase (NadD). This module likely allows the phage to efficiently capture NAD+ precursors from the host cell to support its own replication, and this pathway is one of the means of defense against bacterial antiphage systems, for example, in response to the Thoeris system [2]. NAPR pathway has previously been discovered in dsDNA phages with large genomes exceeding 140 kb, which are myoviruses [3,4].

In addition, the presence of homologues of *the rIIA* and *rIIB genes* from the *RII* locus indicates mechanisms for controlling the timing of lysis and overcoming bacterial abortive infection systems [5,6]. Twenty-two tRNA genes and a tRNA nucleotidyl transferase gene were discovered, which may optimize the translation of viral mRNAs. The genome of phage EC152 has been deposited in NCBI GenBank under the accession number PP681140.1.

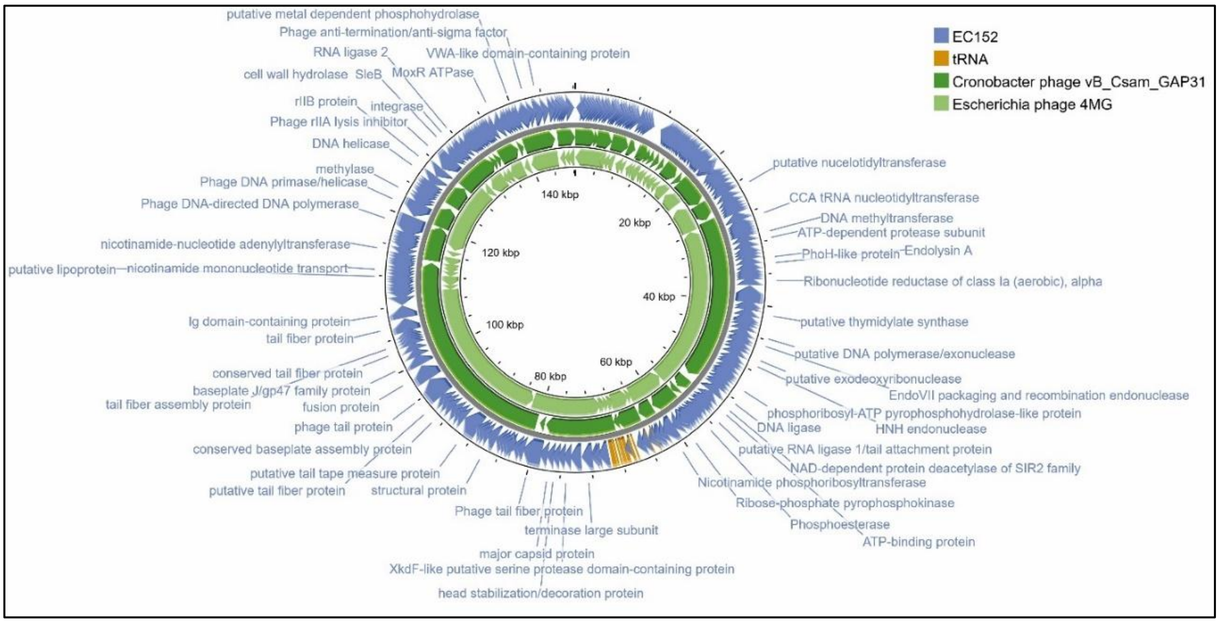

Figure 2EC152 phage genome map constructed using the Proksee server. Open reading frames of the EC152 genome are indicated in blue in the outer circle. TBLASTX was used to compare sequence similarity with *Escherichia phage* 4 MG (light green) and *Cronobacter phage.* vB _ Csam _ GAP 31 (dark green).

Phylogenetic proteomic analysis using ViPTree software revealed that phage EC152 belongs to the genus *Seunavirus,* which includes phages that infect *Salmonella, Escherichia*, and *Cronobacter.* (Fig. 11). The genus *Seunavirus* consists of five phages with genome sizes of approximately ~145 kb and includes the *Salmonella phages* SSE121 (NC_027351), PVPSE1 (NC_016071), and *phage E. coli* 4 MG (NC_022968) and the *Cronobacter phage* vB_CsaM_GAP31 (NC_019400). Although not all phages contain integrase genes (with the exception of vB_CsaM_GAP31), their genomes contain other genes that indicate a temperate lifestyle, such as the genes encoding a transposase-like protein in phages PVPSE1, 4MG, and vB_CsaM_GAP31. Importantly, the genomes of all phages in the genus *Seunavirus* also contain a complete NAD+ utilization pathway.

Thus, phage EC152 is a myophage with lysogenic potential. Genome analysis revealed that it is a member of the genus *Seunavirus* and is the first *Enterobacter* phage described whose genome contains a complete NAD+ utilization pathway.

References:

1. Baker, T.A. Bacteriophage Mu: A Transposing Phage That Integrates like Retroviruses. *Seminars in Virology* **1995**, *6*, 53–63, doi:10.1016/S1044-5773(05)80009-5.
2. Carabias, A.; Montoya, G. NAD Reloaded: Hacking Bacterial Defenses. *Cell Chemical Biology* **2024**, *31*, 1872–1873, doi:10.1016/j.chembiol.2024.10.012.
3. Lee, J.Y.; Li, Z.; Miller, E.S. Vibrio Phage KVP40 Encodes a Functional NAD$^+$ Salvage Pathway. *J Bacteriol* **2017**, *199*, doi:10.1128/JB.00855-16.
4. Li, C.; Liu, K.; Gu, C.; Li, M.; Zhou, P.; Chen, L.; Sun, S.; Li, X.; Wang, L.; Ni, W.; et al. Gastrointestinal Jumbo Phages Possess Independent Synthesis and Utilization Systems of NAD+. *Microbiome* **2024**, *12*, 268, doi:10.1186/s40168-024-01984-w.
5. Ennis, H.L.; Kievitt, K.D. Association of the *r* IIA Protein with the Bacterial Membrane. *Proc. Natl. Acad. Sci. U.S.A.* **1973**, *70*, 1468–1472, doi:10.1073/pnas.70.5.1468.
6. Singer, B.S.; Gold, L.; Shinedling, S.T.; Colkitt, M.; Hunter, L.R.; Pribnow, D.; Nelson, M.A. Analysis in Vivo of Translational Mutants of the rIIB Cistron of Bacteriophage T4. *Journal of Molecular Biology* **1981**, *149*, 405–432, doi:10.1016/0022-2836(81)90479-4.
